# Supplementary material for: Interruption of glucagon signaling augments islet non-alpha cell proliferation in SLC7A2- and mTOR-dependent manners
Source: Mol Metab. 2024 Oct 20;90:102050. doi: 10.1016/j.molmet.2024.102050 (PMC11570739; doi:10.1016/j.molmet.2024.102050)
Supplement: Multimedia component 1 [file mmc1.pptx]

## Slide 1
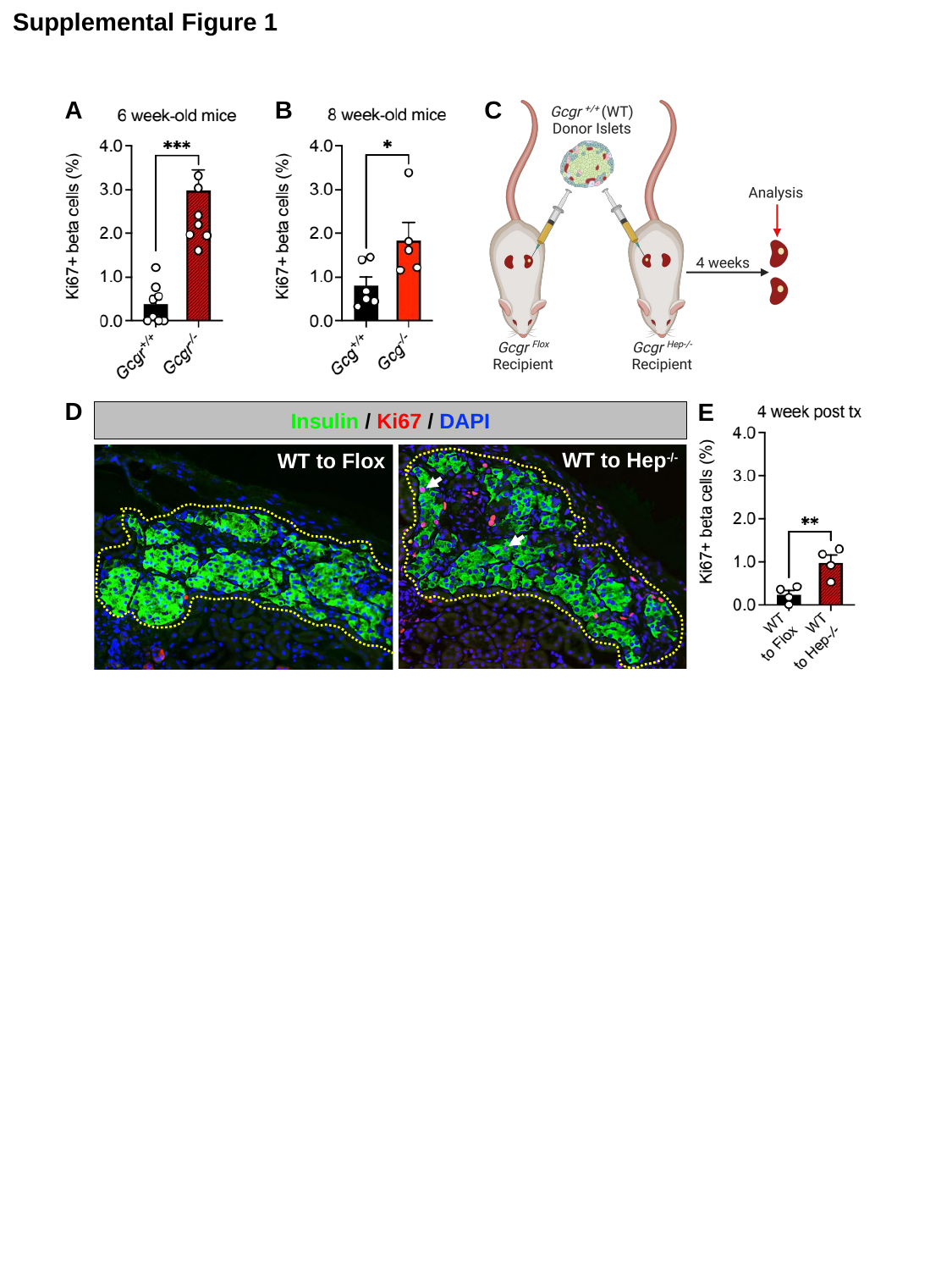

Supplemental Figure 1
A
B
C
D
E
Insulin / Ki67 / DAPI
WT to Hep-/-
WT to Flox

## Slide 2
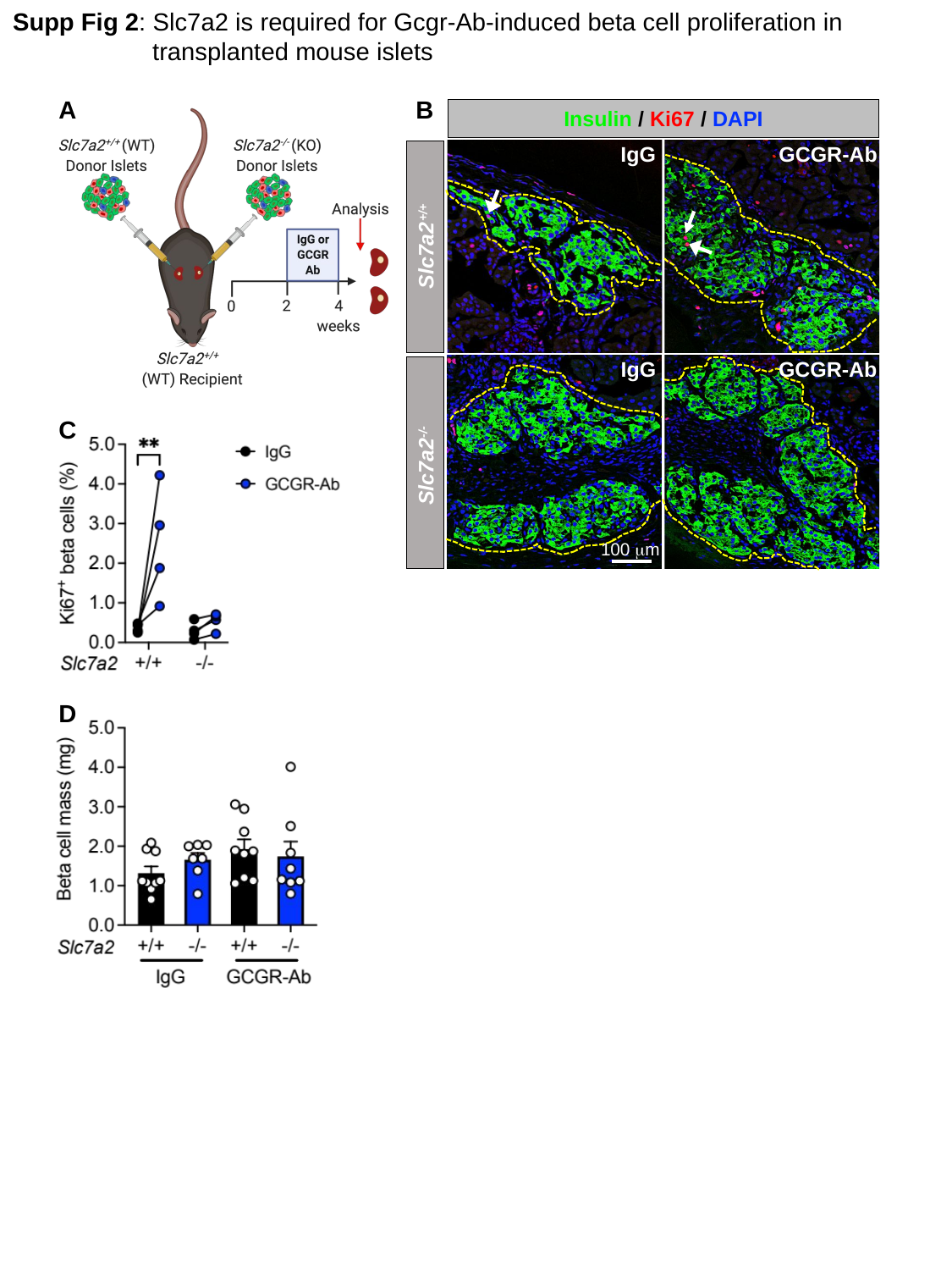

Supp Fig 2: Slc7a2 is required for Gcgr-Ab-induced beta cell proliferation in
 transplanted mouse islets
A
B
Insulin / Ki67 / DAPI
Slc7a2+/+
Slc7a2-/-
IgG
GCGR-Ab
GCGR-Ab
IgG
C
100 mm
D
