# Supplementary material for: Interruption of glucagon signaling augments islet non-alpha cell proliferation in SLC7A2- and mTOR-dependent manners
Source: Mol Metab. 2024 Oct 20;90:102050. doi: 10.1016/j.molmet.2024.102050 (PMC11570739; doi:10.1016/j.molmet.2024.102050)
Supplement: Multimedia component 2 [file mmc2.docx]

Supplemental Figure 1. ***Genetic interruption of glucagon signaling stimulates beta cell proliferation in pancreatic and transplanted mouse islets.*** (**A-B**) Quantification of pancreatic islet beta cell proliferation in (**A**) 6 week-old *Gcgr^+/+^* (black bar, all males) and *Gcgr^-/-^­* (red striped bar, all males) and (**B**) 8 week-old *Gcg^+/+^* (black bar) and *Gcg^-/-^* (red bar) mice (n=2-4 females and 3 males per group, unpaired t test, ***p < 0.001 versus *Gcgr^+/+^*, *p < 0.05 versus *Gcg^+/+^*). (**C**) Schematic of approach for subcapsular renal transplantation of *Gcgr^+/+^* (wild type, WT) donor islets into control (*Gcgr^Flox^*) or liver-specific *Gcgr* knockout (*Gcgr^Hep-/-^*) recipient mice. Created with BioRender.com (**D**) Representative images of islet grafts from WT to Flox and WT to Hep^-/-^ recipients after four weeks. Grafts are immunostained for insulin (green), Ki67 (red) and DAPI (blue). White arrows indicate Ki67+ insulin+ cells. Dashed yellow lines indicate kidney-graft boundary. (**E**) Quantification of beta cell proliferation in transplanted islets from WT to Flox (black bar) and WT to Hep^-/-^ (red striped bar) groups (n=4 males per group, unpaired t test, **p < 0.05 versus WT to Flox).

Supplemental Figure 2. SLC7A2***-dependent*** ***stimulated beta cell proliferation is islet autonomous.*** (**A**) Schematic of approach for subcapsular renal transplantation of *Slc7a2^+/+^* (wild type, WT) and *Slc7a2^-/-^* (KO) 13-15 week old donor islets into 16-18 week old *Slc7a2^+/+^* (WT) recipient mice followed by control IgG or GCGR-Ab 2 week treatment. Created with BioRender.com (**B**) Representative images of *Slc7a2^+/+^* (upper row) and *Slc7a2^-/-^* (bottom row) islet grafts from *Slc7a2^+/+^* kidney capsules after two weeks of IgG or GCGR-Ab treatment. Grafts are immunostained for insulin (green), Ki67 (red) and DAPI (blue). White arrows indicate Ki67+ insulin+ cells. Dashed yellow lines indicate kidney-graft boundary. (**C**) Quantification of beta cell proliferation in transplanted islets from *Slc7a2^+/+^* and *Slc7a2^-/-^* donors treated with IgG (black circles) or GCGR-Ab for 2 weeks (blue circles; n=2 females and 2 males per treatment group, two-way ANOVA with Fisher’s LSD test, **p < 0.01 versus IgG treated). (**D**) Quantification of pancreatic islet beta cell mass in *Slc7a2^+/+^* (black bars) and *Slc7a2^-/-^* (blue bars) IgG or GCGR-Ab-treated mice (n=2-5 females and 3-6 males per group).

Supplemental Table 1: ***Human Islet Donor Information.***

| **Donor ID** | **Age** | **Ethnicity/Race** | **Sex** | **BMI (kg/m^2^)** | **HbA1c (%)** | **Cause of Death** | **Islet Source** |
| --- | --- | --- | --- | --- | --- | --- | --- |
| AELC213 | 10 | Hispanic/Latino | F | 25.4 | N/A | Head Trauma/Blunt Injury | Other |
| AFEA331 | 45 | Black | M | 29.3 | 5.0 | CVA/ stroke | IIDP |
| AIFV371 | 28 | Hispanic/Latino | F | 24.7 | 5.0 | CVA/ stroke | HPAP |
| 1 | 32 | N/A | M | 29.5 | N/A | N/A | IIDP |
| 2 | 47 | N/A | M | 22.3 | N/A | N/A | IIDP |
| 3 | 55 | N/A | M | 28.4 | N/A | N/A | IIDP |
| 4 | 43 | N/A | M | 29.6 | N/A | N/A | IIDP |
| 5 | 46 | N/A | M | 28.8 | N/A | N/A | IIDP |
| 6 | 41 | N/A | F | 31.1 | N/A | N/A | IIDP |
| 7 | 47 | N/A | F | 25.6 | N/A | N/A | IIDP |
| 8 | 52 | N/A | M | 33.2 | N/A | N/A | IIDP |
